# The role of social participation in municipal-level health systems: the case of Palencia, Guatemala

**DOI:** 10.3402/gha.v6i0.20786

**Published:** 2013-09-10

**Authors:** Ana Lorena Ruano

**Affiliations:** Department of Public Health and Clinical Medicine, Epidemiology and Global Health, Umeå University, Umeå, Sweden

**Keywords:** social participation, primary health care, guatemala, alma ata, community participation, community health workers, palencia

## Abstract

**Background:**

Social participation has been recognized as an important public health policy since the declaration of Alma-Ata presented it as one of the pillars of primary health care in 1978. Since then, there have been many adaptations to the original policy but participation in health is still seen as a means to make the health system more responsive to local health needs and as a way to bring the health sector and the community closer together.

**Objective:**

To explore the role that social participation has in a municipal-level health system in Guatemala in order to inform future policies and programs.

**Design:**

Documentary analysis was used to study the context of participation in Guatemala. To do this, written records and accounts of Guatemalan history during the 20th century were reviewed. The fieldwork was carried out over 8 months and three field visits were conducted between early January of 2009 and late March of 2010. A total of 38 in-depth interviews with regional health authorities, district health authorities, community representatives, and community health workers (CHWs) were conducted. Data were analyzed using thematic analysis.

**Results:**

Guatemala's armed civil struggle was framed in the cold war and the fight against communism. Locally, the war was fed by the growing social, political, and ethnic inequalities that existed in the country. The process of reconstructing the country's social fabric started with the signing of the peace agreements of 1996, and continued with the passing of the 2002 legal framework designed to promote decentralization through social participation. Today, Guatemala is a post-war society that is trying to foster participation in a context full of challenges for the population and for the institutions that promote it. In the municipality of Palencia, there are three different spaces for participation in health: the municipal-level health commission, in community-level social development councils, and in the CHW program. Each of these spaces has participants with specific roles and processes.

**Conclusions:**

True participation and collaboration among can only be attained through the promotion and creation of meaningful partnerships between institutional stakeholders and community leaders, as well as with other stakeholders working at the community level. For this to happen, more structured support for the participation process in the form of clear policies, funding and capacity building is needed.

Social participation was first introduced as a pillar of the primary health care (PHC) approach in the Alma-Ata declaration of 1978 (1). The goal of participation was to give people responsibility for their own health by actively engaging them in a process of collaboration with the health sector through the planning and implementation of health policy (2–5). It was through this collaboration that the health system would become more responsive to local needs and become more ‘people centered’ (6).

The declaration states that participation can occur at the community level through community primary health committees or at the individual level by training community members as volunteer health workers (1). Committees act as places where communities and representatives from the health system can work together to discuss and set priorities, to plan the implementation of policies, and to evaluate programs. In places where western trained nurses and doctors are not available, volunteers from the community should be trained as community health workers (CHWs). Today, these are still valid and meaningful ways to participate and interact with the health system (1, 6, 7).

The World Health Organization's (WHO) World Health Report (8) promoted a ‘call back’ to the principles of PHC and the Alma-Ata declaration. Here, the importance of community leadership was once again highlighted and presented alongside a set of health system reforms that strive to make the health policies and health systems more pro-equity. While how participation is defined has changed over the years, it can be described as the process through which individuals or communities interact with the health system (3, 9).

In Latin America, the process of social participation in health has been deeply influenced by the drastic social, economical, and political changes that the region underwent during the second half of the 20th century (3, 10, 11). The economic restructuring policies and the years of political repression and civil war have contributed to creating smaller states incapable of providing adequate health care for their populations (12–14). Many of the countries in the region recognize the importance of participation: Guatemala and Colombia both have wide-ranging but vague legal frameworks to promote it and Brazil has put in place spaces for collective and individual participation that, although not perfect, present an example for neighboring countries (15–17).

Today, social participation in health is enjoying renewed interest because of the WHO's callback to the PHC approach (8). In Latin America, social participation has been part of the mainstream discourse on health for many years and most countries recognize that it is through participation that people can identify their health needs and act on them. According to Almeida (12), participation contributes to the improvement of social inclusion levels and to the development and implementation of pro-equity policy. In Guatemala, social participation is a key component of the decentralization process and an essential part of the health system. The country has a legal framework in place and a national participation scheme to promote participation from the community to the national level. However, little attention has been paid to the process of participation in health and to how it occurs at the municipal and community levels, and to how the experience of community members can influence and shape the process. Because of this, my aim is to explore the role that social participation has in a municipal-level health system in Guatemala in order to inform future policies and programs.

## Background

### Guatemala

This Central American country has a total of 14 million inhabitants, of whom 75% live in rural areas and 52% live in poverty (18, 19). Guatemala is an ethnically diverse country, with 23 indigenous groups that make up about 45% of the population. The other 55% is composed of non-indigenous groups, mostly of mixed European and Amerindian ascent. Life expectancy at birth is 72 years for women and 69 years for men (18). The Gini index of 53.7 shows that there are high levels of inequality in terms of income concentration and distribution, and poverty rates are routinely higher in rural areas than they are in urban ones (19, 20).

Over the course of the 20th century, Guatemala underwent profound social and political changes: the country went from being dominated by liberal dictators through a civil war and finally, through a gradual return to democracy and peace. Until the mid-1940s, the country had liberal, totalitarian governments that concentrated power and resources in the metropolitan area, and that maintained power through the use of political repression, forced disappearances, and torture. A brief 10-year democratic hiatus gave Guatemalans unprecedented social liberties and protections like the abolishment of forced labor and the creation of the social security scheme. However, an armed civil war that lasted from the mid-1950s to the late 1996 left behind more than 200,000 victims of torture, rape, kidnappings, massacres, and murder. Peace was achieved in December of 1996 after the government and the guerrilla came to the agreement that real, everlasting peace could only be achieved through equitable social and economic development for the entire population. Decentralization was to become the main policy for attaining this goal, and social participation was to be the basis of this process. As a result, the state committed itself to developing and creating spaces and frameworks for participation, and to provide the necessary resources for it (21–23). Politically, Guatemala is divided into 22 regions that are subdivided into 333 municipalities. Municipalities act as the organizational and administrative unit of government, and it is at this level that participation is chiefly promoted (24).

The mandate for decentralization is intrinsically linked to ideals of social justice, equality, development, and participation. The process is underlined by the principles of municipal autonomy, the elimination of discrimination and social inclusion (23, 25). In 2002, a legal framework that now included legislation on social development, on a social participation scheme and decentralization, as well as revisions to the municipal code were passed by congress (26). According to the legislation, participation is the involvement of communities and organizations in the planning, execution, and control of the municipal, regional, and national levels of government. Organized communities have the right and the responsibility to audit programs and policies, and the municipality has the obligation to involve its citizens in budgetary processes. This is meant to occur within the country's scheme for participation that goes from the community all the way to the national level (24, 27, 28).

The social development council is a bottom-up structure that constitutes the only officially recognized way to participate in the decentralization process. Participation in these councils is voluntary and there are no economic incentive mechanisms set in place to ensure that community members are involved. According to the council structure, participation begins at the community level and goes through municipal and regional levels all the way up to the national-level council. The scheme created spaces to plan, coordinate, and implement social development policies and means to promote the process of participatory democracy in conditions of equity and equality (24, 25). The national and regional levels of the council offer spaces where civil society and the government officials meet to discuss national-level policies, while municipal- and community-level councils focus more on local issues and priorities. Community councils, made-up of volunteer community members, are to be actively involved in identifying, prioritizing, and solving problems and issues in their own community and municipality. Municipal councils mean to tailor national policies to local needs, to promote inter-institutional cooperation and to be an active part of the development of the budget (24, 25, 27). This level of the council should have specialized commissions committed to education, development, and health among others (27). Although not specified, these committees design plans, projects, and oversee participation from a wide array of stakeholders. However, there are no mechanisms to ensure that the council system works and the law does not specify structures, functions, meeting times, or the way in which the committees should work (25).

### The Guatemalan health system

The health system's resources tend to be concentrated at the central, metropolitan area where only about 25% of the population lives. This leads to very low access to services and higher out of pocket expenditure for the poorest population (29). Although most of the public funds for health are channeled through the Ministry of Health (MoH), investment has declined over the last few years. In addition, the public health system and the social security system offer different schemes of services to different populations, who use them interchangeably depending on the need, availability of care and access (29, 30). The services provided by the public health system are divided into three levels of care: primary care delivered through health centers, health posts, and outsourced non-governmental organizations (NGOs) that provide specific health care packages to rural populations through the extension of care program (29–31).

## Methods

### The municipality of Palencia

Palencia, located in the region of Guatemala, has a population of 55,410 inhabitants of whom 70.3% live in rural areas and 38% are poor (18). Of the total population of this municipality, about 70% have regular access to safe drinking water and electricity; while only 20% have access to sanitation. The main economic activity is subsistence farming and small-scale coffee plantations. There are 49 community-level councils and one municipal-level council within Palencia (19, 32), each of which also has a health commission. The goal of the health commission is to bring the health system and the council scheme together and to provide a space for participation that deals with specific health issues (24).

The municipal-level health commission, which started working in 2009, focuses on the implementation of a health promotion plan that aims to coordinate the work of the municipality, the health center, and the NGO that works under the extension of care program. This plan focuses on reproductive health, a healthy schools initiative, implementation of safe drinking water projects, and prevention of contagious diseases. As a way to test-run the plan, two community-level health commissions that had applied for a project within these main themes were invited to attend the meetings of the municipal-level health commission.

### The health policy triangle framework

**Fig. 1:**
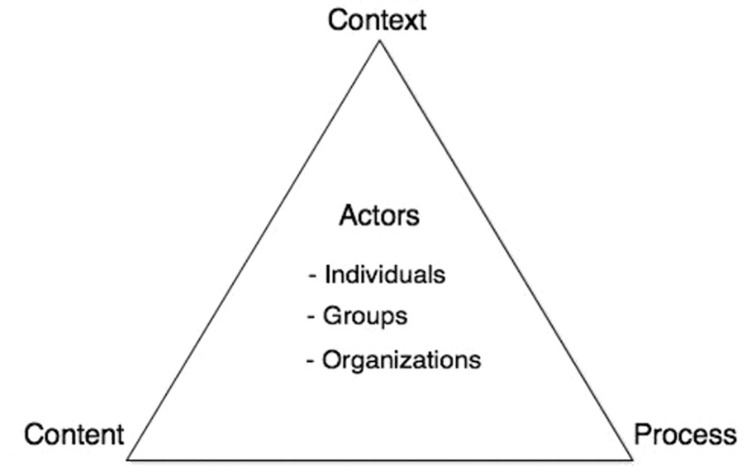
The health policy triangle.

This study used the framework developed by Walt and Gilson in 1994 as a way to explore the process of social participation in Palencia (33). The triangle presented in Fig. 1 shows that there are several components that go into health policy. Health policies are shaped by and contribute to the context, the use of power through influence or resources, and by internal or external factors that might seem unrelated to the policy process itself.

### Applied ethnographic approach

The goal of this approach is to be able to analyze social phenomena through the use of a holistic perspective that can show cultural and social nuances in a way that traditional qualitative or quantitative research cannot. Through its use, I was able to develop praxis-based tools and maintain an open and flexible research design that could address the changing nature of social processes (34–36). I used a combined emic–etic perspective that helped me to collect and analyze a complex and rich narrative to reflect the participants’ social world and the constructs and structures that constitute the process of participation so they could be analyzed in a way that could be useful to policy makers (37, 38). The main method for data collection was participative observation. It allowed me to become familiar with the settings and the participants, and to be a part of meetings, discussions and workshops (39). I also collected data through the use of a camera and a field journal, which provided a way to gather data and track the research process and local culture through my own perspective (36).

As the value of research findings depends on its credibility, ethnographic studies rely on comparability and translatability of research findings instead of the traditional positivistic concepts of generalizability and validity. In order to secure comparability, the characteristics of the groups studied were clearly outlined so that comparisons with similar or different groups could be undertaken later. Translatability assumes that the methods and categories used, as well as the characteristics of the phenomena are explicitly identified so that comparisons can be carried out (37, 38).

### Data collection

The fieldwork for this study was carried out over eight months and three field visits conducted between early January of 2009 and late March of 2010 (with the first visit from early January to the end of March 2009, the second from early June to late August 2009, and the third from early January to late March 2010). During this time, I conducted a total of 38 in-depth interviews with regional health authorities, district health authorities, community representatives, and CHWs. I used semi-structured guides tailored to each stakeholder group with the purpose of gathering rich data regarding the participant's experience and perspectives on the process of participation and the role they played. In addition, I also conducted four semi-structured group discussions with the members of the community-level council in El Triunfo. Most of the interviews were tape recorded and transcribed by me on the same day. For the ones I did not record, notes were taken and later used in the same manner as the transcriptions. All of the observations and informal discussions were completely unstructured and were documented in my field journal through the keeping of expensive field notes written daily at the end of each workday. As advised by Mulhall (39), I only kept one diary that collected my personal experiences and my field notes. This was done in order to analyze my complete field experience later.

### Data analysis

#### Documentary analysis

Documentary analysis aims to analyze written records of accounts through the use of categorized analysis in order to find the key content or main discourse within them and then present these findings in light of larger social and political contexts (35). This method allowed me to cohesively and congruently present data regarding the social, political, and historical context of participation in Guatemala during the 20th century. The first step in this process is to identify sources of information, which was done by reviewing historical documents, personal accounts, and previous work regarding the Guatemalan armed civil conflict. Special focus was put on identifying the impact that the violence levels of this period had on the social fabric of the country as a whole.

The documents used included reports from the UN's Commission on Historical Memory, the Inter-diocesan report for recovery of historical memory. I also reviewed two United Nations Development Program (UNDP) commissioned reports, two peer-reviewed books on the subject and one peer-reviewed paper. These can be found in Refs. 43–49 at the end of the text.

#### Thematic analysis

Thematic analysis is a qualitative method used for discursive interpretation through the finding of recurrent patterns that can be grouped into categories and themes (40, 41). To do this, the data are organized systematically by the identification of topics that link together from codes into categories, and later into meaningful and mutually exclusive themes (41). The data were analyzed manually. The first step in my analysis was to read through all the data, which included data used for the four sub studies that are a part of my doctoral thesis, unused data, and field notes. Because I used the Walt & Gilson framework (33), I already had *a priori* themes selected. However, following the flexibility that the use of the ethnographic approach allowed me, I retained the capacity to add complexity and richness to these themes through emerging categories. The field notes served as contextual information and the photos were used to present richer information in my doctoral dissertation. The last step was to organize and contextualize the themes with a detailed description of the findings (41, 42).

#### Ethical considerations

In Guatemala, it is only necessary to ask for ethical clearance when conducting clinical trials or human testing. However, achieving ethical clearance was of utmost importance so I always presented a detailed plan of activities to all of the stakeholders involved. I also obtained verbal informed consent from all the interviewees. All of the participants were informed that they were free to withdraw from the study at any point and they were all offered anonymity. This request was taken by all the participants except the members of the community-level social development council of El Triunfo. In this case, I used their first names and the real name of their community but did not include their last names. Later, the findings were presented and discussed with the stakeholders in Palencia.

## Findings

### Theme 1: Guatemala; war, peace, and participation

Guatemala's armed civil struggle was framed in the cold war and the fight against communism. Locally, the war was fed by the growing social, political, and ethnic inequalities that existed in the country. Political repression levels remained fairly high for most of the second half of the 20th century, and civil society and community organizations were considered dangerous to the national security (43, 44). By the time the peace agreements where signed in 1996, 25% of the population was deemed to have been a direct victim of the conflict: 200,000 people were murdered or forcibly disappeared, 150,000 children were orphaned and about 1.5 million people from all ethnic backgrounds had been geographically displaced (44). Social and community leaders were specifically targeted. Students, academics, and health workers stationed in rural areas were perceived as especially dangerous (43). This had a negative effect on traditional systems of authority, on community dynamics, and impacted the basic elements of Guatemalan identity (45). Community-level structures were weakened by the use of compulsory mechanisms for participation that tended to replace organic community organization and local groups.

The process of reconstructing the country's social fabric started with the signing of the peace agreements of 1996, and continued with the passing of the 2002 legal framework designed to promote decentralization through social participation. Today, Guatemala is a post-war society that is trying to foster participation in a context full of challenges for the population and for the institutions that promote it. The country still experiences negative consequences from the war, with victims reporting feelings of despair, mistrust and insecurity, and surveys on participation reveal that Guatemalans place very low levels of trust on political and social institutions and public arenas and participatory spaces are perceived as apolitical (46–49).

### Theme 2: framing development through participation

The goal of the legal framework is to create open and structured spaces for participation where different stakeholders can meet, and where local institutions and organized communities can take ownership of policies, programs, and projects. A community-level stakeholder from the municipal-level health commission said:

I think the policies are here to empower the people. It starts with the municipality and then down to us. You could say we're the smallest link in the chain but because of the laws we have a chance to make decisions and ask for solutions to our problems.

Social participation with decentralization policies is perceived as an empowering process that aims to include all different stakeholders in decision making. However, this is not the case when it comes to participation in the health system. One stakeholder from the municipality stated:

[social participation is a way to] … try to get people to not be afraid of going to the doctor, because even when institutions want to work with the communities, our own idiosyncrasies and culture restrict us from accepting care from the physician. The first step in participating is to be open to a medical examination … so that they are aware of their needs.

Community members see participation in health and in decentralization as two activities that belong to the same process. Don José defined participation as:

Grouping together and supporting each other when it comes to health, education and community development. We all have to work together as a team to know where there are faults and what we could do to mend that’.

### Theme 3: organizing participation at the local level

In Palencia, there are three different spaces for participation in health: the municipal-level health commission, in community-level social development councils, and in the CHW program. Each of these spaces has participants with specific roles. In this theme, we explore these three spaces.

#### Organizing participation in health at the municipal level

Palencia's municipal-level health commission has two different types of stakeholders: those that represent institutions such as the municipal government, the health district or health area, and NGOs working in the municipality, and those that represent communities. The first group is better educated and has better access to financial, technical, and human resources, while the community stakeholders have little formal education and balance their work with the commission with their jobs as subsistence farmers.

The institutional stakeholders see themselves as the leaders of the commission, and as the ones that have the power to call meetings in order to attract and engage other stakeholders in this process. The community stakeholders see the commission as an opportunity to present their priorities and to get solutions to their health-related problems. However, institutional stakeholders see this space a one for ‘institutional cooperation’ and community stakeholders lack the power and resources to be part of this process. The legal framework lacks precise and clear instructions on how to coordinate and work with communities and the institutional stakeholders seem unsure on how to do this. One institutional stakeholder reflected:

Well, we have them [the community stakeholders] come so they can be informed, know what is happening. They're meant to listen and well, they have a chance to also voice their opinions and tell us if they agree with what we're doing because we are working in their community and they should also get a say.

#### Continuity in community participation

For the villagers of El Triunfo, community participation is a local tradition that has helped them to carry out large-scale projects such as building a school, a health post and three piped water projects. The council, which is made up of five middle-aged men, has been participating together in different committees and projects for over 30 years. The council members share the value and the belief that participation and helping your neighbor is important. The support they give each other has shaped their experience of participation, and it has contributed to the constancy of their work. Don José said:

We have all gone through this … we have been deputy mayors, council members. The people ask us to it di, they come to us and who are we to say no? So instead of saying no, we say yes … and we sacrifice because it is hard work. It is work we do after we come home. We go to meetings, report on them and act on projects at our expense. Not everyone can do it all the time but we stay … we don't shy away from our responsibility.

During the last three decades, the council members have managed to implement major projects in collaboration with municipal, national, and international stakeholders and donors. Their volunteer work has changed the community where they live. So far, it has been difficult to involve other community members in the process because most are worried about the extra workload and are scared they will make mistakes. Don Roberto shares his ideas for carrying on this tradition of community participation:

People think that if you make a mistake everyone will laugh at you but you just have to learn and keep going. It is tough to go to a meeting and not know anything but you pick up things along the way and that helps … José thinks you have to get involved and you can see his two-year-old grandson is always here. He sees everything that we do and learns … so it's only a matter of time until he starts doing this too!

#### Being a CHW

The program to extend care has created a space for individual participation at the community level through its CHW program, where villagers can work closely with their neighbors and with the doctors and nurses from the health team. This has given the CHWs access to learning opportunities and many of them reported finishing grammar school or health promoter classes as a result of their work with the CHW program. Esmeralda reported only having basic reading skills and being forced out of school at an early age. Her work gave her the opportunity to learn new skills. She said:

I like this job because we learn new things, we learn them in a clear way so that stuff that used to be blurry, things we only used to hear others talk about, we understand now … it makes me happy to know these things and to work with them.

Participation and community involvement are family traditions in Palencia, and many CHWs reported having active parents, siblings, or spouses. The shared values of community service and volunteerism encourage people to participate and the position of CHW provides a space to put these values into practice. When this is combined with the support, they feel from the health system and the visible improvements in their communities, it creates a sense of commitment that helps them deal with negative aspects of their work. As Doña Aura said:

You feel the responsibility to serve because it starts like something you volunteer for and then you see it as something ‘you have to do’. It's like a duty and you just have to do it. That motivates you, to see it like a responsibility, so that when you come home from your normal work and are tired you keep going. It's the sense of responsibility that keeps you going.

### Theme 4: reflections on a process

People in Palencia can participate at different levels and in different spaces, and while their responsibilities and roles might differ, they share the commitment to improve the quality of life of their neighbors through their actions. In this theme, we present three reflections: one from a council member in El Triunfo, one from a CHW and one from an institutional stakeholder.

Don Miguel, from El Triunfo:

There is no time to lose. First you have your family and then your community. This is true at the social as well as at the religious level … our motivation comes from our needs and the enthusiasm and interest we have in improving our family and everyone else's lives. And when people say ‘let's do it’ we feel good, like when neighbors help each other. The motivation is our community. If no one else stands up, you have to … when you stop paying attention to the community's lack of support, you start improving your community.

Doña María, a CHW who is now recognized as a community leader:

People look up to me because once they saw me as a leader they always try to get me involved in everything. I give the community information and help in any way that I can. No problem for us to do it, to help a little.

The people in Palencia see the importance of promoting participation in health, even if at times they are unsure of how to do it. An institutional stakeholder said:

Like everywhere, there are some people that participate and some that do not. What we need is more communication about what we are doing, what we hope to do and what we need to do it.

## Discussion

Today, the Guatemalan state recognizes that democracy and strong institutions can only come through fair and equitable development and the participation of the people. The challenge is to repair the torn social fabric that resulted from the country's violent history and the systematic social and political exclusion of ethnic and rural population face (22, 48). The villagers in Palencia saw very little of this violence and for them, community involvement and public life continued to be viewed positively. However, the geographical isolation that kept the Palencian countryside safe also meant that there were very few chances for economic growth opportunities during the 20th century.

It has been 15 years since the signing of the peace agreements and 10 years since the passing of the legal framework that highlights the role that communities have in the social and economic development process of the country. Guatemala is now a democratic and relatively stable country that is able to guarantee basic political rights. However, the return to democracy and the end of the war were processes promoted by Guatemalan elites that sought to take power from the guerrilla and the army, and not the result of organic, grassroots movements (50). As a result, the country continues to have weak institutions with thin institutional culture that are unable to disperse power consistently among stakeholders (51). In addition, the lack of concrete guidelines when it comes to how to implement participation processes hinders the Guatemalan process. In contrast, Brazil's participation schemes have a clear structure with easy to follow rules that guarantee access to special federal funds. As a result, Brazil's system has a means to balance out the personal interests of more powerful stakeholders (52, 53). Because the Guatemalan legal framework is vague, participants interpret it based on their abilities, agendas, and on the resources they have. This leaves each municipality free to have their own version of what participation in health means.

The three spaces of participation presented here reflect different stages of the policy cycle, from participation in planning to the implementation and evaluation of policies. The municipal health commission is a relatively new space where participants derive their power from their ability to bring resources for coordination to the table and the goal is to create institutional collaboration. Although the community-level stakeholders lack the power resources to be a part of this established collaboration, their view tends to reflect the spirit of inclusion and dispersion of decision-making power that is at the heart of the policies (51). Their perspective is not in conflict with the institutional stakeholders, who are also coping with the social, political, and policy-related changes that are impacting their traditional way of working.

The issue of unbalanced power relations can leave community-level stakeholders feeling disenchanted with the participation process (54). However, all of the stakeholders in Palencia report feeling positive about the process and similar to everyone are participating to the extent that their time and financial capabilities afford them. This is because for the institutional stakeholders, the commission is an additional responsibility that taxes their already-loaded workweek. They lack the time, tools, and incentives to be a part of the commission and the possibility to coordinate their already scarce resources is what keeps them at the meetings. The community stakeholders cannot attend all the meetings due to financial and time constraints, but the results they have obtained clearly outweighs their negative feelings over their low levels of decision-making power.

The individual- and community-level participation processes presented here are better established and have longer trajectories thanks to previous experiences with community participation and health promoter projects. Both of these processes have benefited from Palencia's geographical isolation, which protected the villages from the destructive effect that the armed internal conflict had in other parts of Guatemala (3). This allowed for the continuation of community practices, values, and institutions.

El Triunfo's social development council has been able to maintain a stable participation process that has engaged local, national, and international stakeholders thanks to the contextual and personal elements that serve as the backbone to their work. Solidarity and a desire to serve are at the core of the work the council members do. The deep bond between them comes from being part of a tight-knit community where the villagers feel connected to each other through their work, values, family bonds, and religious beliefs (55). The group's identity is based on the desire to improve the life of everyone in the community and is grounded in the deep friendship these men share, along with the sense of accomplishment that comes from reaching long-standing goals (56).

Palencia's CHWs have developed a tight social network that has been able to link their communities to the health system through their work in a way that has provided deep personal satisfaction (57). This happened by recognizing the role that volunteerism can play in improving community life. Having close family members who are also active provides the CHWs with support, guidance, and motivation from people that recognize the importance of the work they do (7). The bonds that exist between the CHWs and the health team have been developed organically and are based on mutual respect, on the interaction of their personalities, and on shared values. This has contributed positively to their feelings of satisfaction and creates opportunities for personal growth, which also plays a role in keeping the CHWs engaged in their everyday work.

The experience of participation in health is understood through the building of common meanings, the sharing of values, and by the motivation each individual has in the context of the work they carry out every day (58, 59). The participants of these three spaces have been able to establish processes based on self-reliance, equity, social justice and community protection, values that are also central to the PHC approach (1, 60). Through the sharing of the core values of the Alma-Ata declaration, the actions that participants carry out have a deeper meaning and provide a deep level of satisfaction. The embodied experience of participation results in the people in Palencia seeing their work as a tool that helps them stay involved in the larger process of community development (61).

The process of participation provides more than just a platform for community members to express their values through tangible actions in their villages. Through it, they have the opportunity to learn new skills, and this has contributed to provide satisfaction when circumstances such as lack of recognition, payment or severe resource constraints could have deterred their participation.

To understand the impact of participation in health, we must analyze the process alongside with how participating has influenced the lives of the people involved. From the outside perspective, Palencia shows a wide range of participation practices that can go from simple information or cost offsetting to ones where community members are equal partners with more powerful stakeholders (62). From the insider perspective, we see that participation can have a deeper meaning that cannot be analyzed simply by its outward results. If community participation is seen as a manifestation of shared values then the outcomes of a process go beyond that of a policy (61). The experiences presented here show that participation can lead to personal growth and that the work that the people in Palencia carry out on a daily basis provides deep satisfaction and personal gratification (62, 63).

The richness of the findings lie in the qualitative data obtained from 8 months of participant observation on situated practices at the municipal, community, and individual levels. Through the daily interactions with the participants of this study, I was able to present the human perspective of each of these practices while framing them in the overall social, political, and historical context of the country. However, I was aware that it is impossible to cover every aspect of a social process. As my interest was to study the experience of social participation in health, I did not use a health system approach that could have focused on how effective or efficient these practices were. Palencia is a municipality that was not hit by the decades of armed civil conflict, its population is predominantly non-indigenous and villages have been geographically stable for many years. This makes Palencia different from other parts of Guatemala. However, I content that while the context of many other parts of the country is strikingly different, there are lessons to learn from the long-running participation practices that can be found at the community level and municipal level. Finally, while I did not set out to not consider issues of gender and participation, this is the path the fieldwork took. Issues around gender balance in Guatemala, and specifically in Palencia, require further studies.

### A model for understanding social participation in health

**Fig. 2:**
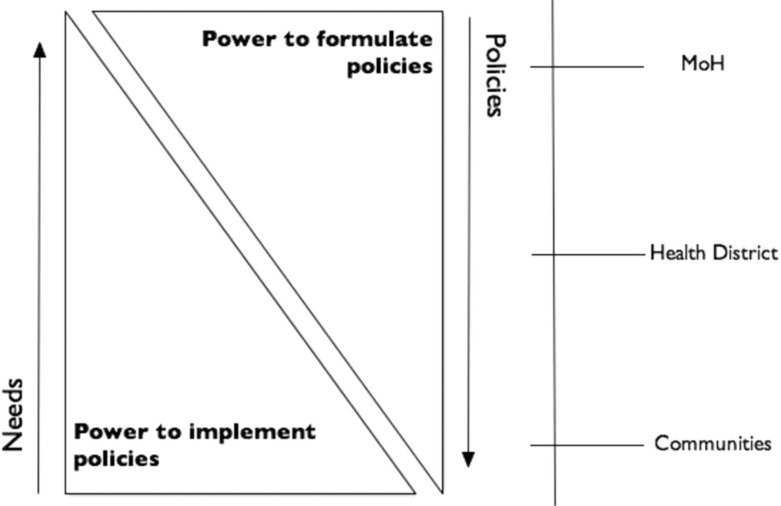
Participation, power, and health policy: a model for understanding stakeholder's roles and collaborations in public health policy.

In Guatemala, social participation is the main policy behind the ongoing decentralization process, and it is backed by a legal framework and a participation scheme that aims to create open and democratic spaces for participation. This process can be shown through the model presented in Fig. 2, which shows how different roles and power resources that each stakeholder has are related to the activities and the level of involvement they have in the process.

The figure refers to the process of participation in Palencia, where one of the key activities of the social development councils is the identification of needs at the local level. The arrow shows how these needs travel up the participation structure and are discussed at the community, district, and national levels. The discussions inform policy makers, who then relay polices back down the structure where they are shaped to fit the specificities of the context. The model presents how these two activities are intrinsically linked to two kinds of power, that is, power to formulate policy and power to implement it.

These two kinds of power are complementary but depend on the amount and the kind of resources available at each level of the participation structure. Stakeholders with power to formulate policies will have better access to financial, human and material resources and information, while stakeholders with higher levels of power to implement policies have resources such as community legitimacy, knowledge of local culture, values and mores, and a deep understanding of local social processes. These different powers help to balance the structure of participation and remind stakeholders of the abilities that lie outside of them, making the role of collaboration more important and visible and highlights that in these kinds of participation processes, no stakeholder is truly powerless.

The municipal-level commission is seen as a space for institutional collaboration for the coordination of scarce resources, and the institutional stakeholders tend to have power to formulate and to implement policies. However, by not including more community stakeholders in the process of discussing and making policy decisions the commission is not using all of the available resources. The coordination of financial and human resources is just as important as the legitimacy that comes from having community leaders involved in more steps of this process, and this can only be obtained through meaningful partnerships between institutional and community members and other stakeholders that are working on the ground.

The council from El Triunfo shows that meaningful participation can result in achieving long-standing community goals. Through their 30 years of participation, these men have been able to achieve long-standing community goals. The foundation for this process is the deep bonds of friendship that these men share, as well their individual and shared values. The council members have been able to mange their low levels of power to formulate policies to plan social development projects while using their high levels of policy implementation power to ensure their implementation and sustainability over long periods of time. At the individual level, the CHWs have established deep and meaningful bonds with their communities, bonds that are based on shared values with neighbors, co-workers, and people from the health center. The participation process has allowed them to learn new skills and the recognition from the work has made many of them community leaders. This has positive implications for the CHW program and has strengthened the relationship between the communities and the health system.

## Conclusion

Social participation is a complex process that can provide enriching experiences that can lead to personal growth, to the fulfillment of long-standing community goals and to the improvement of collaborations between stakeholders. In order for these processes to be successful, they need clear frameworks and policies that reward involvement at the national, district and community levels. Community participation processes need strategic support from outside stakeholders and enough time for them to develop into legitimate institutions such as the council in El Triunfo. Finally, when it comes to individual participation, a combination of intrinsic motivation based on personal values and beliefs combined with encouragement from outside stakeholders can empower community members. What is needed now is more structured support for these processes in the form of policies, funds, and capacity building.
